# Biosynthesis and Toxicological Effects of Patulin

**DOI:** 10.3390/toxins2040613

**Published:** 2010-04-05

**Authors:** Olivier Puel, Pierre Galtier, Isabelle P. Oswald

**Affiliations:** INRA, UR66 Pharmacologie-Toxicologie, F-31027 Toulouse, France; Email: pgaltier@toulouse.inra.fr (P.G.); ioswald@toulouse.inra.fr (I.P.O.)

**Keywords:** patulin, biosynthesis, polyketide, toxicity

## Abstract

Patulin is a toxic chemical contaminant produced by several species of mold, especially within *Aspergillus*, *Penicillium* and *Byssochlamys*. It is the most common mycotoxin found in apples and apple-derived products such as juice, cider, compotes and other food intended for young children. Exposure to this mycotoxin is associated with immunological, neurological and gastrointestinal outcomes. Assessment of the health risks due to patulin consumption by humans has led many countries to regulate the quantity in food. A full understanding of the molecular genetics of patulin biosynthesis is incomplete, unlike other regulated mycotoxins (aflatoxins, trichothecenes and fumonisins), although the chemical structures of patulin precursors are now known. The biosynthetic pathway consists of approximately 10 steps, as suggested by biochemical studies. Recently, a cluster of 15 genes involved in patulin biosynthesis was reported, containing characterized enzymes, a regulation factor and transporter genes. This review includes information on the current understanding of the mechanisms of patulin toxinogenesis and summarizes its toxicological effects.

## 1. Introduction

Patulin was first isolated by Birkinshaw *et al.* [1] in 1943 from *Penicillium griseofulvum* and *Penicillium expansum*. This was part of the screening effort to find new fungal molecules with antibiotic properties, in the general enthusiasm following the discovery of penicillin by Fleming. Patulin fits well with Paracelce’s definition in his treatise “Von der besucht”. "Every substance is a poison; only dose distinguishes a poison from drug". This compound was tested in clinical trials by a British company under the brand name “tercinin” [2], however, the interest in this potential antibiotic soon waned due its toxicity to humans and animals.

Today, patulin belongs to a short list of mycotoxins (aflatoxins, ochratoxin A, zearalenone, fumonisins and trichothecenes) whose level in food is regulated in many countries around the world, with European countries being among the first to propose limits in the levels. Since 2003, European regulation 1425/3003 sets a maximum level of 50 µg/L for fruit juices and derived products, 25 µg/L for solid apple products and 10 µg/L for juices and foods destined for babies and young infants [3]. Today, the US Food and Drug Administration (FDA) limits patulin to 50 µg/L.

Patulin is isolated from several species belonging to *Penicillium*, *Aspergillus*, *Paecilomyces* and *Byssochlamys*. A recent exhaustive review echoes the old studies that reported patulin production by a large number of patulin producing species related to not less 30 genera [4]. Several studies based on analysis of secondary metabolites by HPLC-DAD (High Pressure Chromatography coupled with Diode Array Detector) or LC-MS (Liquid Chromatography coupled with Mass Spectrometry) have allowed revision of the number of patulin producing species.

Among the *Aspergillus* species, the number of patulin producing species is limited to three of the Clavati group: *Aspergillus clavatus*, *A. giganteus* and *A. longivesica* [5].

For the *Penicillium* genus, after checking a significant number of isolates from each species and re-identification of certain isolates, a recent overview determined 13 patulin producing species: *P. carneum*, *P. clavigerum*, *P. concentricum*, *P. coprobium*, *P. dipodomyicola*, *P. expansum*, *P. glandicola*, *P. gladioli*, *P. griseofulvum*, *P. marinum*, *P. paneum*, *P. sclerotigenum*, *P. vulpinum* [6].

In the case of the *Paecylomyces* and *Byssochlamys*, two independent research groups demonstrated recently that *B. fulva*, formerly identified as patulin producers, do not produce this mycotoxin [7,8]. A comparative study of all *Byssochlamys* and related *Paecilomyces* species carried out using a polyphasic approach showed that only *B. nivea* and some strains of *Paecilomyces saturatus* produce patulin [9]. 

**Figure 1:**
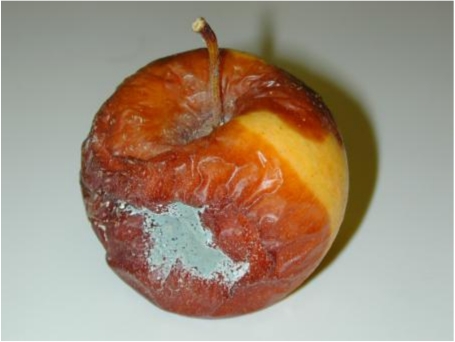
An apple contaminated by *Penicillium expansum*.

Among these species, *P. expansum* is responsible for the decay in pomaceous fruits (apples and pears) characterized by rapid soft rot and eventually by blue pustules (Figure 1). This species is considered as the main source of patulin in these fruits and consequently in apple derived products [10].

Here, we review past research devoted to the understanding of the mechanisms of patulin biosynthesis in fungi, and we summarize the effects of this toxin on hosts that ingest it.

## 2. Biosynthesis of Patulin

### 2.1. Precursors and enzymatic activities

**Figure 2:**
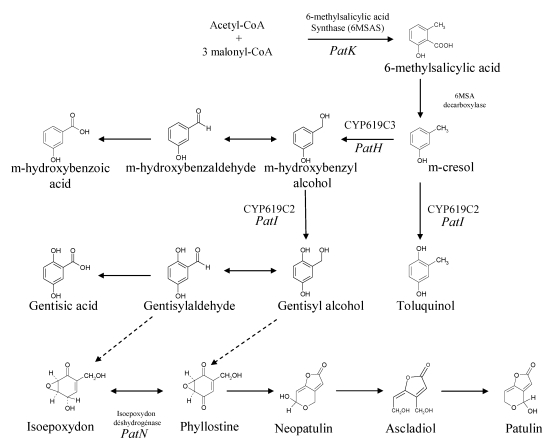
Scheme of patulin biosynthetic pathways. Adapted from [29,41,107].

Patulin is a polyketide metabolite, like several other major mycotoxins, e.g., aflatoxins, fumonisins and ochratoxins, although this last toxin is a polyketide/amino acid hybrid compound. The study of its biosynthesis is historically significant for two reasons. Firstly, the acetated hypothesis of Birch [11] was based upon the incorporation of radiolabeled acetate into 6-methylsalicylic acid (6MSA), and led to the recognition of a major class of natural products, the polyketides, previously suggested by Collie. The second reason is that the enzyme involved in the first patulin biosynthesis step was the first polyketide synthase to be studied and characterized *in vitro* [12]. The biosynthetic pathway of patulin consists of about 10 steps as suggested by several biochemical studies and by the identification of several mutants that are blocked at various steps in the patulin biosynthetic pathway [13] (Figure 2). 

The first step in the production of patulin is the formation of 6MSA by the condensation of one acetyl-CoA and three malonyl-CoA units. This formation is carried out by a single multifunctional enzyme that has several enzymatic activities: acetyl and malonyl transferase, ketoacyl synthase, ketoreductase and dehydratase [12]. This enzyme also possesses an acyl carrier protein function [14,15] and consists of four identical 176 kDa polypeptidic chains [16]. Historically, Bu’Lock and Tanenbaum showed that radiolabeled 6MSA was converted into patulin [17,18].

The studies using ^14^C and ^3^H radioisotopes and ^13^C and^ 2^H stable isotopes showed that 6MSA is then modified extensively to form patulin. The products generated from *m*-cresol and gentisylaldehyde are structurally similar to 6-methylsalicylic acid [19,20,21]. 6MSA is modified to *m*-cresol by 6MSA decarboxylase, then the methyl group of *m*-cresol is oxidized to form an aldehyde group. This step is followed by a hydroxylation reaction that leads to gentisaldehyde formation. However, at this point, it seems that for over a decade the sequence of the different intermediates was much less clear. The conversion of gentisaldehyde to a two ring structure such as patulin needs the opening of a ring by a mechanism mediated either by a monooxygenase or by a dioxygenase. The isolation of several patulin-minus mutants of *P. griseofulvum* led to the identification of four post aromatic precursors: isoepoxydon [22] phyllostine [23], neopatulin [24] and ascladiol [25]. The discoveries of these compounds led also to the hypothesis that an epoxidation step occurs after gentisaldehyde synthesis. The nature of the epoxidation has remained a matter of speculation since a point of contention existed as to whether the substrate of the epoxidation reaction was gentisyl alcohol or gentisaldehyde [26]. Indeed, although a kinetic pulse labeling study performed by Forrester and Gaucher [19] showed that only the following co-metabolites are readily converted into patulin: acetate, 6MSA, *m*-cresol, *m*-hydroxybenzyl alcohol, *m*-hydroxybenzaldehyde and gentisaldehyde, a crude extract which catalyzed the epoxidation of gentisyl alcohol to phyllostine was isolated [26]. 

Several enzyme activities related to patulin biosynthesis have been characterized. Among them, the ring hydroxylation of *m*-hydroxybenzyl alcohol to gentisyl alcohol requires NADPH and molecular oxygen for activity. Inhibition in a reversible manner by carbon monoxide suggests the involvement of cytochrome P450 [27].

Five of the enzymes involved in patulin biosynthesis have been partially or fully purified: the first, 6-methylsalicylic acid synthase, the second, 6-methylsalicylic acid decarboxylase [28], m-hydroxybenzyl alcohol dehydrogenase, the seventh, isoepoxydon dehydrogenase (IDH) [29] and the eighth, neopatulin synthase [29].

### 2.2. Patulin gene cluster

In filamentous fungi, the genes encoding the enzymes involved in the synthesis of secondary metabolites are usually contained together in clusters on chromosomes [30,31]. A large number of gene clusters related to secondary metabolite production has been discovered and particularly those responsible for the biosynthesis of several mycotoxins, e.g., aflatoxins [32], fumonisins [33], trichothecenes [34], ergot alkaloids and zearalenone [35]. In contrast, a gene cluster involved in patulin biosynthesis has only recently been isolated and only some genes related to patulin biosynthesis have been identified. The first gene was the 6-methylsalicylic acid synthase (6MSAS) gene from *P. griseofulvum* [14,15]. The *idh* gene encoding the seventh enzyme, isoepoxydon dehydrogenase (IDH), was originally isolated from *P. griseofulvum* [29], and then from several other *Penicillium* species [36,37,38] and from *B. nivea* [8,39]. Recently, a gene located downstream of the *idh* gene has been isolated, which encodes a protein with a high homology to isoamyl alcohol oxidase [40]. Finally, genes encoding cytochrome P450 enzymes—involved in two steps of the patulin biosynthesis pathway—have been isolated from *A. clavatus*, and characterized by heterologous expression in yeast [41]. The first cytochrome P450 (CYP619C3) transforms *m*-cresol to yield *m*-hydroxybenzyl alcohol whereas the second cytochrome P450 (CYP619C2) catalyses the hydroxylation of *m*-hydroxybenzyl alcohol to gentisyl alcohol. This last monooxygenase can also hydroxylate *m*-cresol to 2.5 dihydroxytoluene (toluquinol). This result confirms an earlier experiment showing that the same enzyme preparation could convert *m*-cresol and *m*-hydroxybenzyl alcohol respectively to toluquinol and gentisyl alcohol [26,27]. Toluquinol is a well-known compound produced by *P. griseofulvum*. Its role as patulin precursor was discussed in earlier studies but the use of a pulse–chase radiolabeling technique or a culture of force-fed deuterated toluquinol has shown that toluquinol is metabolized to deoxyepoxydon and not to patulin [19,20]. On the basis of these studies, toluquinol is a co-metabolite of patulin, but not an intermediate in the patulin biosynthetic pathway.

Recently, a cluster of 15 genes involved in patulin biosynthesis has been identified in the *A. clavatus* genome [41]. All the genes are located in a 40 kb region. The genes encode the enzymes necessary for the biosynthesis of the toxin, but also the specific regulatory factor and transporters. This cluster contains three transporter genes: one ABC (ATP binding cassette) transporter, one MFS (Major Facilitator Superfamily) transporter and one acetate transporter. The cluster also contains genes for enzymes: one putative carboxyl esterase (*PatB*), one putative Zn-dependent alcohol dehydrogenase (*PatD*), one GMC (Glucose-Methanol-Choline) oxidoreductase (*PatE*), one gene that probably encodes a putative decarboxylase displaying an amido hydroxylase conserved domain (*PatG*), two genes (*PatH* and *PatI*) encoding cytochromes P450 (responsible for the hydroxylation of *m*-cresol to *m*-hydroxybenzyl alcohol and of *m*-hydroxybenzyl alcohol to gentisyl alcohol, respectively) and a gene encoding a putative dioxygenase (*PatJ*).

**Figure 3:**
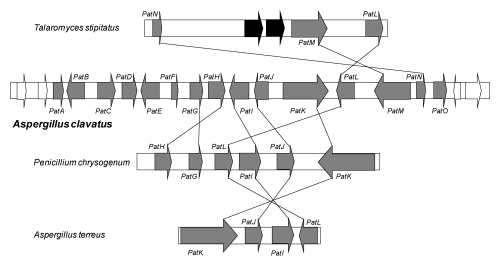
Patulin gene cluster in *A. clavatus* and comparison of secondary metabolite gene clusters in three other fungi species that contain some genes related to patulin production. Grey indicates genes of the patulin cluster; white indicates genes adjacent to the patulin cluster. The black arrows in *T. stipitatus* represent genes that are not present in the patulin gene cluster.

The cluster contains also the *6msas* gene (*PatK*), one putative transcription factor gene (*PatL*), the isoepoxydon dehydrogenase gene (*PatN*) previously isolated as *idh* gene, a putative isoamyl alcohol oxidase described recently from *P. griseofulvum* (*PatO*), and finally a gene which does not have any obvious function (*PatF*). Related clusters are also present in the genome of some species that do not produce patulin such as *P. chrysogenum*, *Talaromyces stipitatus* and *Aspergillus terreus*. They are also shown in Figure 3. The fact that these latter species do not produce patulin can be explained by the lack of some key genes such as the *6msas* gene in *T. stipitatus* or *idh* gene in *A. terreus* or *P. chrysogenum*. Although Varga, *et al.* [42] confirmed that *A. terreus* was not a patulin producing species, the isolation of the *AtX* gene encoding a 6MSAS has been previously reported. The gene encoded a functional enzyme that led to 6MSA formation after heterologous expression in *A. orizae* [43]. In *A. terreus*, another biosynthesis pathway, that of terreic acid, needs 6-methylsalicylic acid synthase activity [44]. Since there is only one *6msas* gene in the *A. terreus* genome, it is very likely that this gene encodes an enzyme involved in terreic acid biosynthesis in this species.

Although their roles are not proven, the involvement of some genes can be predicted with regard to the steps identified biochemically in the synthesis of patulin. PatG contains the amido hydroxylase superfamily signature sequence motifs shared by γ-resorcylate decarboxylase and 5-carboxyvanillic acid decarboxylase [45]. Pat G is most likely involved in the decarboxylation of 6-methylsalicylic acid to *m*-cresol. On the basis of the high homology with VBS (59%), which catalyses the transformation of versiconal into versicolorin B in the aflatoxin pathway, and the similarity between their chemical structures, PatE could catalyze the last step of the patulin pathway, to yield patulin from ascladiol. The very recent demonstration that vesicles play a key role in aflatoxin biosynthesis and the hypothesis of the role of subcellular compartmentalization could be explain the presence of three transporter genes in the cluster [46].

### 2.3. Regulation of patulin biosynthesis

The level of nutrient nitrogen in the culture medium determines when the patulin pathway is expressed. In their study, Grootwassink and Gaucher [47] showed that the age at which a *P. griseofulvum* culture produced the key enzyme *m*-hydroxybenzyl alcohol dehydrogenase increased when the nutrient nitrogen content of the medium increased or when the size of the inoculums decreased. Resuspension of the mycelium in a nitrogen-free 4% glucose solution immediately induces the pathway enzymes and patulin production. Distinct metabolic phasing can also be observed in cultures with ammonium as the sole nitrogen source. When ammonium ions were added to cultures actively producing patulin, a rapid loss of secondary metabolism occurred [48]. The effect of ammonium repression has been determined to be at the transcription level. The addition of 30 mM ammonium chloride to cultures producing *idh* and *6msas* transcripts caused a considerable decrease in the amounts of both transcripts, suggesting that a rapid turnover of patulin mRNA occurred and not enzyme inhibition. This down-regulation has been also observed for various mycotoxins such as sterigmatocystin and ochratoxin A [49,50]. In *P. griseofulvum*, the upstream regions of both the *6msas* and *idh* genes have several putative GATA sites that could interact with a global nitrogen regulatory factor encoded by the *nrfa* gene, an *AreA* orthologous gene in *P. griseofulvum*. In a reporter gene study, Summerer [51] showed that reporter gene activity is observed only in cultures lacking ammonium and that deletion of regions containing the proposed GATA binding sites resulted in a loss of expression activation. DNA mobility shift assays demonstrated that the upstream regions of the 6MSA synthase and isoepoxydon dehydrogenase genes bind strongly to the NRFA DNA binding domain [52]. 

In contrast, there is no carbon metabolite repression of patulin biosynthesis. Like aflatoxin, glucose is an optimal carbohydrate source for patulin production but it is unclear if the carbon source directly induces patulin production or modulates its biosynthesis through general metabolism.

Manganese had been shown to be an essential requirement for patulin biosynthesis in *P. griseofulvum* [53]. Inhibitor studies using actinomycin D and cycloheximide showed that manganese exercised its affect on patulin biosynthesis at the level of transcription and influenced the coordinated appearance of pathway enzymes [54]. This was confirmed by Northern Blot analysis [29]. Manganese has a substantial effect on the expression of the *idh* gene, but only a limited effect on the *6msas* gene, resulting in 6-methylsalicylic acid accumulation but very little patulin production.

In *P. griseofulvum* shaken cultures, the 6MSAS enzyme seems to appear four hours before the 6MSA decarboxylase, *m*-hydroxybenzylalcohol dehydrogenase, and isoepoxydon dehydrogenase. Induction of later enzymes in the pathway by certain patulin pathway intermediates has been shown [55]. Indeed, the addition of early pathway metabolites *i.e.*, 6MSA or *m*-hydroxybenzyl alcohol to 16 hour old shaking cultures shifted the appearance of the fourth enzyme forward by about two hours. When various concentrations of 6MSA or *m*-hydroxybenzyl alcohol were added to growing cultures of a mutant strain unable to produce patulin and 6MSA, the second enzyme, 6MSA decarboxylase appeared 1.5 hours earlier, with maximum levels about seven-times higher than the uninduced control. Induction was greatest with *m*-hydroxybenzyl alcohol. This regulation has been shown to occur at the transcriptional level [29].

Finally, using a reporter gene strategy, Summerer [51] showed that the pH of the culture medium affected the transcription of the *idh* gene. In *P. griseofulvum*, optimal transcription occurs when the initial culture pH is 5.0, and decreases when the pH is either raised or lowered.

## 3. Toxicity of Patulin

### 3.1. General toxicity

Patulin has a strong affinity for sulfhydryl groups. Patulin adducts formed with cysteine are less toxic than the unmodified compound in acute toxicity, teratogenicity, and mutagenicity studies. Its affinity for SH-groups explains its inhibition of many enzymes.

### 3.2. Acute toxicity

In rodents, the oral LD50 of patulin ranges between 29 and 55 mg/kg body weight (b.w.) [56,57,58]. Poultry seem less sensitive, with an oral LD50 of 170 mg/kg b.w. When administered by the intravenous, intraperitoneal or subcutaneous routes, patulin is 3-6-times more toxic. Toxic signs consistently reported in all studies were agitation, in some cases convulsions, dyspnea, pulmonary congestion, edema, and ulceration, hyperemia and distension of the gastro intestinal tract [59].

Some compounds were able to modulate the toxicity of patulin. For example, inhibitors of cytochrome P450 such as proadifen (SKF 525A) considerably increase the toxicity of this mycotoxin, while P450 inducers do not modify its toxicity [60]. Similarly, when a patulin/cysteine adduct was administered to mice intraperitoneally, no acute toxicity was observed at levels up to 150 mg of patulin/mouse [61].

### 3.3. Sub-acute toxicity

The sub-acute administration of patulin has been mainly studied in rats, where it was shown to induce weight loss, gastric and intestinal changes and alterations in renal function [62,63]. Repetitive doses lead to signs of neurotoxicity (tremors, convulsions) as well as an inhibition of several enzymes (ATPase) in the intestine [64] and the brain, in particular, with consequences in terms of lipid metabolism [65].

Similar clinical signs were observed in mice, hamsters and chickens. In monkeys, no sign of toxicity was observed after daily treatments with 5 to 500 µg/kg b.w. for four weeks. Only monkeys receiving 5 mg of patulin/kg b.w./day for two weeks rejected their food during the last three days of the experiment [66]. 

Selmanoglu and Kockaya [67] measured thyroid and testicular hormones in rats receiving 0.1 mg patulin/kg b.w./day patulin by the oral route for 60 or 90 days. A 60-day exposure increased the plasma level of testosterone and decreased T_4_ hormone while there was no change in T_3_, TSH, LH and GH. When the exposure lasted for 90 days, there was an increase in testosterone and in LH without any other clinical signs. Histological examination of the thyroid showed lymphoid cell infiltration and enlargement of interstitial tissue. At the tested level, edema, fibrosis, local Leydig cell hyperplasia and disorganization of the seminiferous tubule epithelium were observed. This was associated with a decreased sperm count [68]. 

As summarized in Table 1, patulin is recognized as mainly inducing gastrointestinal disorders with ulceration, distension and bleeding, and at higher doses, alterations in renal function.

### 3.4. Genotoxicity

Patulin did not increase revertant frequency in the Ames test using several strains of *Salmonella* *typhimurium*, but some studies have shown mutagenic activity in *Saccharomyces cerevisiae* strains and in *Bacillus subtilis* [69,70]. Patulin gave a negative result for genotoxicity by the SOS microplate assay [71], however, it was clearly positive in the initiator tRNA acceptance assay for carcinogens [72].

**Table 1:** Summary of sub-chronic studies describing the effects of patulin.

Species	Dose	Duration	Observations	Reference
Mice	24–36 mg/kg b.w. every day or every other day	14 days	Intestinal disorders	[56]
Rat	28–41 mg/kg b.w. every day or every other day	14 days	Intestinal disorders	[58]
Rat	25–295 mg/L in drinking water	28 days	Decreased weight	[62]
			Decreased Cl creatinine	
			Gastric ulcers with high doses	
Rat	0.1 mg/kg b.w. every day	30 days	Decreased lipids	[65]
			Decreased triglycerides	
			Increased cholesterol	
			Inhibition of intestinal ATPase	
Rat	6–150 mg/L in drinking water	13 weeks	Decreased food intake	[63]
			Decreased weight with high doses	
Rat	0.1 mg/kg b.w. every day	60 & 90 days	Increased testosterone and LH levels	[67]
			Alteration of testis and thyroid morphology	
Rat	0.1 mg/kg b.w. every day	60 & 90 days	Decreased sperm count	[68]
			Alteration in sperm morphology	
Hamster	16 mg/kg b.w. every day or every other day	14 days	Intestinal disorders	[57]
Chicken	100 µg every other day	30 days	Intestinal disorders	[64]
			Alteration of renal function	
			Inhibition of intestinal and renal ATPases	
Monkey	5; 50; 500 µg/kg b.w. then 5 mg/kg b.w. every day	30 days 45 days	No toxicity	[66]
			Food refusal (high dose)	
			Alteration of renal function (medium dose)	

Several studies have demonstrated that patulin acts as a clastogen in mammalian cells, e.g., inducing micronuclei without kinetochores and structural chromosomal aberrations in cultured Chinese hamster lung fibroblast V79 cells [73,74,75]. Chromosome aberrations and gene mutations were induced by patulin in FM3A cells, a C3H mouse mammary carcinoma cell line [76], in V79 cells [77], and in mouse lymphoma L5178Y cells [78]. Chromosome and chromatid gaps and breaks were also induced by patulin in Chinese hamster ovary cells but not in human peripheral blood lymphocytes [79]. Similar differences in the susceptibility of different cell systems to the genotoxic activity of patulin were reported with respect to sister chromatid exchanges, which were induced in Chinese hamster ovary cells and human peripheral blood lymphocytes [79] but not in Chinese hamster V79 cells [73]. Evidence for oxidative DNA damage was obtained in human embryonic kidney cells after treatment with patulin [79,80] and it is also implied by the observation that ascorbic acid decreases the induction of micronuclei and chromosomal aberrations in PAT-treated V79 cells [75].

In conclusion, although the data on genotoxicity were variable, most assays carried out with mammalian cells were positive while assays with bacteria were mainly negative. In addition, some studies indicated that patulin impaired DNA synthesis. These genotoxic effects might be related to its ability to react with sulfhydryl groups and to induce oxidative damage [80,81]. Nevertheless, the WHO [59] concluded from the available data that patulin is genotoxic.

### 3.5. Cancerogenicity

The few studies of long-term toxicity on patulin showed an absence of tumors in rats orally exposed to 0.1 to 2.5 mg patulin/kg b.w./day for 74 to 104 weeks [82,83]. According to the International Agency for Research on Cancer (IARC), patulin is classified in the group 3 as ‘‘not classifiable as to its carcinogenicity to humans” [70].

### 3.6. Embryotoxicity and teratogenicity

As part of a two-generation reproduction study, the offspring of Sprague Dawley rats were exposed to 1.5 mg patulin/kg b.w./day [84]. Patulin caused an increased resorbtion in F1 litters, but this effect was not observed in the F2 generation. In this generation only a decrease in the weight of the foetuses without observable deformations was noticed. Similar observations were made by Reddy, *et al.* [85] in rats receiving 1.5 mg patulin/kg b.w. intraperitoneally; a dose of 2 mg toxin/kg b.w. induced the abortion of all embryos.

In mice, the same oral dose provoked the death of the offspring with brain, lung and cutaneous haemorrhages [82]. A higher oral dose (3.8 mg/kg b.w./day for 12 days) did not modify the number of implantations and delivered fetuses without inducing any deformation. By contrast, intraperitoneal injection increased cleft palate as well as malformations of the kidneys [86]. 

When injected into the air cell of chick eggs, patulin was reported to be embryotoxic at levels of 2.35–68.7 µg/egg depending on the age of the embryo, and teratogenic at levels of 1–2 µg/egg. Patulin/cysteine adducts showed the same toxic effects, but at much higher doses: 15–150 µg of patulin equivalents [61]. Rat embryos were exposed to patulin-treated (0.00–62 µM) rat serum for 45 h. The embryos that were exposed to the highest dose did not survive beyond 40 h of incubation. Patulin induced a significant reduction in the protein and DNA content, yolk sac diameter, crown rump length, and somite number count. Patulin treatment also increased the frequency of defective embryos. Anomalies included growth retardation, hypoplasia of the mesencephalon and telencephalon, and hyperplasia and/or blisters of the mandibular process [87].

### 3.7. Immunotoxicity

As described for other mycotoxins, patulin can alter the immune response of the host [88]. Numerous *in vitro* studies have demonstrated that patulin inhibits several macrophage functions. Indeed, Sorenson *et al.* [89] showed that *in vitro* exposure of alveolar rat macrophages to patulin inhibited protein synthesis and altered membrane functions. Patulin also significantly decreased the production of O_2_^−^, phagosome-lysosome fusion, phagocythosis, as well as lysosomal enzyme and microbiological activity in mouse macrophages [90]. 

More recently, patulin was found to reduce the cytokine secretion of IFN- γ and IL-4 by human macrophages [91] and of IL-4, IL-13, IFN-γ, and IL-10 by human peripheral blood mononuclear cells and human T cells [92]. However, this mycotoxin was less potent than gliotoxin [91]. Similarly, Marin *et al*. [93], using the thymoma cell line EL-4, observed a reduction in the production of interleukins 2 and 5, with concentrations of 500 ng patulin/ mL. Similarly, in this study, the inhibitory effect of patulin was lower than that observed with T-2 toxin. This decrease in cytokine secretion was not due to the cytotoxic effects of patulin but to the depletion of intracellular glutathione [92]. 

*In vivo* studies using mice indicate variable effects of patulin on the immune system. These effects include an increased number of splenic T lymphocytes and depressed serum immunoglobulin concentrations [94], depressed delayed hypersensitivity responses [95] and increased neutrophil numbers and resistance to *Candida albicans* infection [96].

A thorough battery of immunology endpoints was examined in mice administered patulin daily by gavage for 28 days at doses of 0.08 to 2.56 mg patulin/kg b.w. [97]. These doses were calculated to be approximately equivalent to estimated human exposure levels. Changes in immune cell numbers included depressed peripheral blood leukocytes and lymphocytes numbers (observed at 1.28 and 2.56 mg toxin/kg b.w./day), an increased number of splenic monocytes and Natural Killer cells (from 0.08 mg toxin/ kg b.w./day), an increased number of cytotoxic T lymphocytes (at 2.56 mg patulin/kg b.w./day) and changes in the percentage of immunoglobulin (Ig)^+^, CD3^+^, CD4^+^/CD8^− ^and CD4^−^/CD8^+^ lymphocytes in the spleen. These changes in cell number did not reflect functional changes. There were no measurable changes in immune function in patulin-treated mice using the following endpoints: antibody response to sheep red blood cells (SRBC), mixed leukocyte responses and Natural Killer cell functions. The authors concluded that exposure to patulin, at levels consistent with potential human exposure in food would not be likely to alter immune responses [97].

In rats, as in other species, patulin reduces the oxidative burst in peritoneal macrophages [93] and the production of reactive oxygen species in HL-60, human human promyelocytic leukemia cells [80]. Mc Kinley *et al.* [58] suggested that the increase in neutrophils was due to gastro-intestinal inflammation induced by the toxin.

## 4. Conclusion

In recent years, only a few studies have been published on the *in vivo* toxicity of patulin. Most of the toxicological studies have used *in vitro* models and focussed on the immunotoxic and genotoxic effects of the toxin. Recent studies have also demonstrated that patulin alters the intestinal barrier function [98,99]. Indeed, when contaminated food is ingested, the intestine is the first organ in contact with mycotoxins [100] and intestinal epithelia cells are targets for these toxins [101,102].

When compared with the biosynthesis of aflatoxins and trichothecenes, the understanding of the molecular basis of patulin toxinogenesis is in an early stage. Indeed, the gene cluster has only been isolated recently. Although, most of the patulin intermediates have been characterized structurally, the role of the majority of the genes located in the patulin gene cluster is still unclear. Several studies have shown that many environmental factors control patulin biosynthesis, and thus we can anticipate that many layers of regulation are involved in patulin biosynthesis as already demonstrated for the regulation of aflatoxin biosynthesis. For example, the involvement of PatL, located in the patulin cluster, in the regulation of patulin production should be confirmed. Similarly, several studies have shown that the regulatory elements elements LaeA and VeA are involved in the regulation of mycotoxins [103,104,105,106], but in the case of patulin this still needs to be demonstrated.

Despite the efforts to reduce the patulin levels in all stages of the apple product processes, the occurrence of this mycotoxin is still high throughout the world. A better understanding of patulin biosynthesis would help to limit patulin contamination during the storage of apples in cold rooms.
